# Polatuzumab Vedotin联合利妥昔单抗和苯达莫司汀治疗复发/难治性弥漫大B细胞淋巴瘤的单中心疗效和安全性分析

**DOI:** 10.3760/cma.j.issn.0253-2727.2022.01.013

**Published:** 2022-01

**Authors:** 繁聪 孔, 敏 喻, 玉兰 周, 诗轩 王, 菲 李

**Affiliations:** 南昌大学第一附属医院血液病诊治中心，南昌大学淋巴肿瘤疾病研究所，南昌 330006 Center of Hematology, Institute of Lymphoma of Nanchang University, The First Affiliated Hospital of Nanchang University, Nanchang 330006, China

弥漫大B细胞淋巴瘤（DLBCL）占非霍奇金淋巴瘤（NHL）的31％～34％[1]，部分患者接受R-CHOP（利妥昔单抗+环磷酰胺+阿霉素+长春新碱+泼尼松）方案一线治疗可治愈，但仍有30％～40％的患者复发或难治，特别是活化B细胞型（ABC）、双表达或双打击淋巴瘤等[2]。复发/难治性（R/R）DLBCL患者中位总生存（OS）期仅为6个月[3]–[4]，治疗尚无统一标准，含吉西他滨和（或）铂类药物等二线方案、自体造血干细胞移植（auto-HSCT）和新型靶向药物等均可作为挽救性治疗选择。近年来，嵌合抗原受体修饰T细胞（CAR-T细胞）疗法成为治疗R/R DLBCL的重要手段，但细胞制备周期长、费用高及细胞因子释放综合征等限制了其临床应用[5]–[6]。因此，临床上仍然亟需寻找有效的新型药物和治疗手段。

CD79b是一种B细胞特异性表面蛋白，表达于90％的B-NHL。Polatuzumab vedotin（Pola）是一种靶向CD79b蛋白的抗体偶联药物，包括靶向CD79b的重组人源化IgG1单抗、可裂解型linker（连接桥）及小分子药物MMAE（单甲基澳瑞他汀E）。Pola与肿瘤细胞的CD79b结合后抑制细胞分裂并诱导细胞凋亡，实现对细胞的靶向杀伤作用[7]–[8]。2019年，一项全球Ⅰb/Ⅱ期临床研究显示Pola联合BR（苯达莫司汀+利妥昔单抗）方案治疗R/R DLBCL患者的完全缓解（CR）率为40％（对照组BR方案组的CR率为18％）[9]。基于此，Pola获得美国食品药品监督管理局（FDA）突破性疗法认定，并被加速批准用于治疗既往已接受至少2种疗法的R/R DLBCL患者[10]。但Pola在我国尚未获批上市，本研究通过分析总结本中心R/R DLBCL患者应用Pola的临床数据评估其疗效及安全性，为中国R/R DLBCL患者使用该方案提供临床依据。

## 病例与方法

1. 入组病例：Pola同情用药项目（compassionate use program，CUP）得到南昌大学第一附属医院医学伦理委员会批准，伦理批号：伦临审2019第164号。入组标准为：①诊断为 R/R DLBCL，并经组织病理学确诊；②既往已接受至少二线治疗方案，如R-CHOP方案（或与DLBCL一线治疗方案相似的方案，如R-DA-EPOCH、R-mini-CHOP、R-EDOCH、R-EPOCH、R2-COP方案等）；③不适合接受allo-HSCT或auto-HSCT；④既往未接受过BR方案治疗；⑤未发生≥2级周围神经病变（peripheral neuropathy，PN）。复发或难治定义为在接受标准或常规治疗后<6个月内发生疾病进展（PD）或疾病稳定（SD），或接受治疗后缓解≥6个月后疾病复发。共纳入我院2020年3月1日至2021年3月31日符合Pola CUP入组条件的11例患者，所有患者均签署了书面知情同意书。

2. 治疗方案：纳入分析的11例患者均使用Pola+BR方案。具体用药剂量如下：利妥昔单抗375 mg/m^2^，第1天，静脉输注；Pola 1.8 mg/kg，第2天，静脉输注；苯达莫司汀90 mg/m^2^，第2、3天，静脉输注。每21 d为一个周期。下一疗程给药前如出现以下情况需进行剂量调整：①≤3级输注反应，中断Pola输注并予支持治疗。如首次出现喘息、支气管痉挛、全身性荨麻疹等3级或4级输注反应，需永久终止Pola用药。②中性粒细胞绝对计数（ANC）<1×10^9^/L或PLT<75×10^9^/L，延迟所有用药。若中性粒细胞和血小板恢复时间超过1周，苯达莫司汀剂量逐次递减为70 mg/m^2^、50 mg/m^2^，若苯达莫司汀已减至50 mg/m^2^，则降低Pola剂量至1.4 mg/kg。③PN：对于2～3级PN，暂停Pola直至恢复至≤1级PN，若恢复时间不超过2周，Pola剂量降至1.4 mg/kg，如既往已降至1.4 mg/kg或恢复时间超过2周则终止Pola用药；4级PN需永久终止Pola用药。

3. 疗效和生存分析：参照2014版Lugano分类标准[11]进行疗效评估，临床主要观察指标为CR、部分缓解（PR）、SD或PD。次要观察指标包括OS期、无进展生存（PFS）期和缓解持续时间（DOR）。所有患者在治疗的第1、3、5个疗程前以及第6个疗程结束后通过增强CT或PET-CT检查评估疗效。

4. 安全性评价：观察并记录患者在治疗期间出现的各种不良反应（AE）。AE分度参照美国国家癌症研究所不良事件通用术语标准（NCI CTCAE）5.0版本进行。安全性评估包括AE、严重AE（SAE）和特别关注的AE（AESI）。本研究中的AESI定义为血小板减少≥3级、中性粒细胞减少≥3级、贫血≥3级、PN≥2级、感染≥3级。

5. 统计学处理：采用SPSS 25.0软件进行数据分析，分类资料用例数描述，计量资料用中位数（范围）描述。生存分析采用Kaplan-Meier法。

## 结果

1. 患者基线临床特征：共纳入11例已接受至少2个疗程Pola+BR方案并进行了至少1次疗效评估的患者，其中男6例，女5例；中位年龄60（20～75）岁；既往中位接受4（2～7）线治疗。Ann Arbor 分期Ⅰ、Ⅱ、Ⅲ、Ⅳ期患者分别为2例、0例、2例、7例；美国东部肿瘤协作组体能状态评分0分1例，1～2分10例；生发中心来源（GCB）患者5例，非生发中心来源（non-GCB）患者6例。双表达患者4例（定义为病理组织免疫组化MYC表达>40％，BCL-2表达>50％）。所有患者在既往治疗方案中均使用过利妥昔单抗，9例患者既往使用过包括PD-1单抗、HDAC抑制剂、BTK抑制剂或BCL-2抑制剂等药物的治疗。2例患者既往接受过靶向CD19的CAR-T细胞治疗，其中1例患者接受CD19和CD22双靶点CAR-T细胞治疗。1例患者为原发耐药（定义为一线治疗中对治疗无反应，包括SD及PD）。

2. 治疗：共11例患者接受了Pola+BR联合治疗，其中1例患者因经济原因在第3、4个疗程未用苯达莫司汀。4例患者因出现血小板减少2级且持续时间超过1周，苯达莫司汀减量为70 mg/m^2^；1例患者因血小板减少4级且恢复时间超过2周，苯达莫司汀先后减量为70 mg/m^2^、50 mg/m^2^。所有患者Pola未发生剂量调整。

3. 疗效：截至2021年4月30日，4例患者结束了全部6个疗程治疗；4例患者由于PD分别在第2个疗程后（1例）、第4个疗程后（3例）更换治疗方案；1例患者第3个疗程结束后因血小板减少恢复时间长退组，更换治疗方案；1例患者在第3个疗程结束后因粒细胞缺乏并发严重感染死亡而终止治疗；1例患者已结束3个疗程治疗，目前仍处于治疗中。所有患者的疗效及生存情况见图1。11例患者的最佳疗效分别为：CR 1例，PR 8例，SD 1例，PD 1例，总反应率（ORR）81.8％。

**图1:**
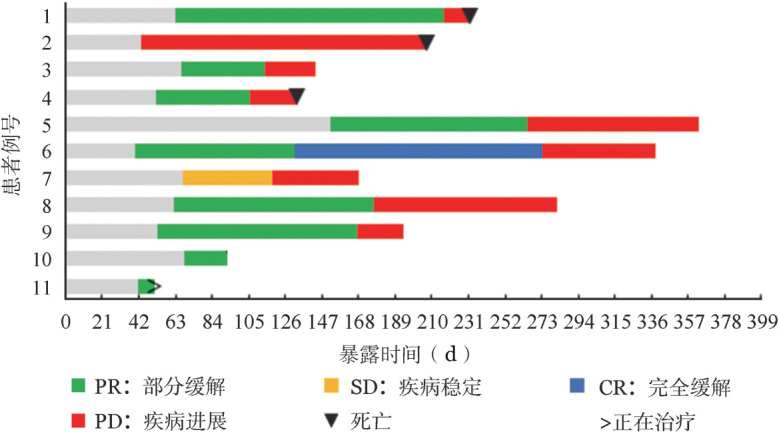
11例复发/难治性弥漫大B细胞淋巴瘤患者的疗效及生存情况

4. 生存分析：截至2021年4月30日，中位随访时间9.5（1.7～13.5）个月，存活病例7例，其中5例在随访期间发生PD，1例疾病复发，目前均更换为其他方案，1例患者仍处于治疗进程中。4例患者死亡，其中3例因PD死亡，1例因粒细胞缺乏并发严重感染死亡。11例患者中位OS期、PFS期分别为未达到（NR）和7.5个月，9例获得PR以上疗效患者的中位DOR为5.0个月。

5. 安全性：大部分患者对Pola+BR方案耐受性良好。8例患者发生了至少1种AE，大部分为血液学不良反应，经对症治疗或观察等待后均可恢复，5例患者因AE调整药物剂量。最常见的AE为：血小板减少（5例）、贫血（3例）、中性粒细胞减少（2例）、恶心（2例）。5例发生血小板减少的患者中4例程度为2级，经升血小板药物治疗后血小板恢复正常；1例发生4级血小板减少，此例患者因血小板恢复时间长而退组，被认定为AESI。2例患者发生中性粒细胞减少，1例1级，1例2级，经对症治疗或观察等待后恢复。1例患者发生发热性中性粒细胞减少，此例患者在第3个疗程治疗结束后因粒细胞缺乏并发严重感染而死亡，该事件被认定为SAE。3例贫血患者均为2级。其他较少出现的AE为：PN（1例）、嗜睡（1例）、乏力（1例），均为1级。1例患者发生3级肺部感染，经抗感染治疗后恢复。值得注意的是，1例患者在治疗过程中出现噬血细胞综合征，被定为SAE，经过积极治疗后患者达CR，至今存活。

## 讨论

CUP是指对患有严重或危及生命疾病的患者，在无法通过现有药品或进入临床试验获得有效治疗时，医师可在临床试验以外申请使用试验药品的制度。近年来，同情用药在欧盟国家陆续开展并建立法律依据。随着我国医疗健康保障水平的提升及医药产业发展，在我国建立同情用药制度具有迫切的临床和社会价值[12]–[13]。作为在中国开展的CUP，Pola CUP使R/R DLBCL患者在疾病进展阶段能得到及时、有效的治疗，并为中国R/R DLBCL患者使用Pola联合BR方案提供初步的疗效和安全性数据。

目前全世界范围内开展了多项关于Pola治疗R/R DLBCL患者的临床研究。Sehn等[9]的研究表明，Pola+BR组与BR组相比，ORR为45.0％对17.5％、CR率为40.0％对17.5％（*P*＝0.026）、PR率为5.0％对0、SD率为15.0％对2.5％，BR组更低，而PD率为20.0％对25.0％，Pola+BR组更低。与BR组相比，Pola+BR组的死亡风险降低了58％，患者PFS期（9.5个月对3.7个月，*P*<0.001）和OS期（12.4个月对4.7个月，*P*＝0.002）明显延长。在接受Pola+BR方案治疗的患者中，48％（12/25）的患者治疗反应持续时间至少1年，而应用BR方案的患者治疗反应持续时间达1年者仅占20％（2/10）。中位随访30个月，约20％应用Pola+BR方案治疗的患者维持无进展状态[14]。

R/R DLBCL患者后续治疗缓解率低。一项回顾性研究分析了636例R/R DLBCL患者的临床资料，治疗后ORR为26％，CR率仅7％，中位OS期为6.3个月[15]。本研究中，Pola+BR方案治疗11例R/R DLBCL患者的ORR为81.8％，CR率9.1％，PR率72.7％，中位随访9.5个月，中位OS期、PFS期、DOR分别为NR、7.5个月、5.0个月。本中心入组患者均为临床难治患者，经Pola+BR方案治疗后短期疗效显著。

截至末次随访日期，大部分存活患者（6/7）均发生PD或复发，提示该方案短期疗效好，但长期疾病控制率有限，患者达到PR及以上疗效后需积极寻求其他治疗手段，如CAR-T细胞治疗或造血干细胞移植以获取最佳生存。因此Pola+BR方案可作为后续治疗的有效桥接治疗手段。

Pola+BR方案AE主要表现为血液学AE、PN和胃肠道反应[10]。血液学AE主要为血小板减少、贫血、中性粒细胞减少及发热性中性粒细胞减少。在Pola单药或Pola联合利妥昔单抗方案的临床试验中，3～4级中性粒细胞减少发生率分别为4.0％和23.0％。Pola联合BR治疗时，3～4级中性粒细胞减少发生率升高为46.0％[16]。Pola+BR组与BR组相比，发热性中性粒细胞减少发生率相似（23.1％对20.5％），而所有级别贫血和血小板减少的发生率较高（分别为46.7对28.2％和48.9对33.3％）[17]。本研究中1～2级中性粒细胞减少发生率为18.2％，无3～4级中性粒细胞减少，3～4级血小板减少发生率约为9.1％。本研究中仅观察到1例患者出现1级PN，1例患者发生噬血细胞综合征。总体上，本组患者血液学和非血液学AE比例与国外报道相比并未增加，Pola+BR方案用于R/R DLBCL患者总体安全性和耐受性良好。

作为回顾性真实世界研究，本研究受到以下因素制约：①Pola CUP入组标准为既往二线以上治疗失败的患者，本研究纳入患者既往接受的中位治疗线数为4（2～7）线，因此治疗难度相对更高；②1例患者因经济原因在第3、4个疗程未联合使用苯达莫司汀，评估的是2个疗程的疗效，既往研究表明Pola+R两药联合方案的疗效劣于Pola+BR三药联合方案[14]；③部分患者在治疗中因PD和AE退出，虽然ORR为81.8％，但因随访时间短，无法准确评估DOR；④样本量偏小。由于存在上述缺陷，该结果不适合与Sehn等[9]国外研究结果进行直接比较。

总之，中国关于Pola治疗R/R DLBCL患者的临床研究数据较少，本研究数据来自中国第一个Pola CUP，结果证实Pola+BR方案用于R/R DLBCL患者的安全性和耐受性均良好，对R/R DLBCL患者不失为一种有效治疗选择，特别是对于既往接受过多疗程、多线治疗的复发难治患者，能获得明显的短期疗效，延长PFS期，为桥接后续治疗赢得时间和机会。由于纳入样本量偏少，随访时间短，未来需纳入更多患者以获得长期疗效、安全性和生存数据。
